# Editorial: Complement in nervous system disease

**DOI:** 10.3389/fncel.2023.1268023

**Published:** 2023-08-07

**Authors:** Iliana Michailidou, Kees Fluiter, Marina Boziki, Nikolaos Grigoriadis, Frank Baas

**Affiliations:** ^1^Laboratory of Experimental Neurology and Neuroimmunology and the Multiple Sclerosis Center, 2nd Department of Neurology, AHEPA University Hospital, Aristotle University of Thessaloniki, Thessaloniki, Greece; ^2^Department of Clinical Genetics, Leiden University Medical Center, Leiden, Netherlands

**Keywords:** complement, immunity, neuroinflammation, neurodegeneration, therapy

Complement is an ancient system for host anti-microbial aid with a major role in the remodeling of the developing nervous system. It serves the innate immunity, interfaces with the adaptive immunity (Ricklin et al., 2016; Reis et al., 2019), and mediates the elimination and refinement of synapses (Stevens et al., 2007; Schafer et al., 2012). It consists of more than 30 proteins which circulate in the serum and have an effector or regulatory role. Complement proteins are mainly produced by the hepatocytes (Zhou et al., 2016) and cannot enter the nervous system in the presence of an intact blood-brain/spinal cord barrier. However, some components are produced within the nervous system (Morgan and Gasque, 1997) to mediate developmental processes (Magdalon et al., 2020) or neuroimmune responses (Veerhuis et al., 2011; Michailidou et al., 2015, 2017).

Complement recognizes non-cognate antigens and devours “unwanted” cells or cell compartments (Ricklin et al., 2010; Nonaka and Nakanishi, 2019). Activation of complement occurs via three pathways, the classical, lectin, and alternative pathway (Figure 1). C1q is the initiator of the classical pathway. It binds to immune complexes or to “eat me” signals exposed by apoptotic cells and it has an important role in the pathogenesis of both neurological (Dalakas et al., 2020) and non-neurological (Coss et al., 2023) diseases. A systematic review by Schulz and Trendelenburg provides a comprehensive overview of all the studies published between 1998 and April 2022 which utilized the C1qKO mouse to induce experimental models, providing information on the molecular contribution of C1q to human diseases.

**Figure 1:**
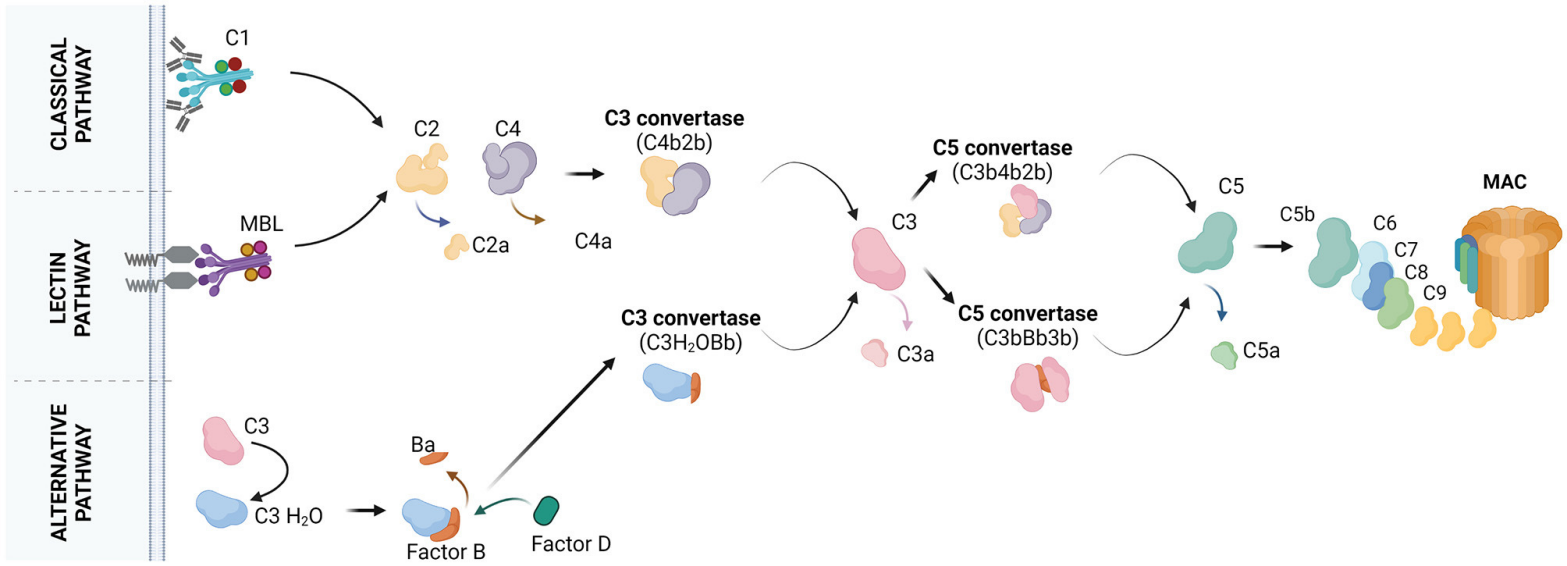
The molecular pathways of complement activation. Classical pathway: it starts with the binding of C1q to the target cell. C1q recognizes the target by the presence of bound antibodies. C1q can also bind to distinct molecules, patterns or structures. C1q is a subunit of the C1 complex which is further composed of the C1r and C1s proteases. Upon binding of C1q to the target, C1r and C1s get activated. C1s cleaves the C4 protein into C4a and C4b. C4a is released whereas, C4b is covalently bound to the target. C4b attracts C2 which is cleaved by C1s into C2a and C2b. C2a is released whereas, C2b binds C4b forming the C4b2b complex, also known as C3 convertase. C3 convertase cleaves C3 into C3a and C3b. C3b may bind the C3 convertase forming the C4b2b3b complex, also known as C5 convertase. C5 convertase cleaves C5 into C5a and C5b. C5b may associate with C6, C7, C8, and C9 to form the final product of complement activation, the membrane attach complex (MAC). Lectin pathway: it starts with the binding of mannose-binding lectin (MBL) or ficolins on the target cell. Circulating MBL or ficolins recognize the target by the presence of carbohydrate patterns. MBL is complexed with two components, the MBL-associated serine protease (MASP) 1 and MASP2. MASPs get activated upon binding of MBL to the target and cleave C4 and C2 to form the C3 convertase. The pathway continues with the formation of the C5 convertase and MAC. Alternative pathway: it starts with the spontaneous hydrolysis of C3 into C3(H_2_O) and continues with the binding of factor B to C3(H_2_O) for cleavage of factor B into Ba and Bb by factor D. This results in the formation of the fluid phase C3 convertase, C3(H_2_O)Bb. C3(H_2_O)Bb converts C3 into C3a and C3b. Some C3b molecules attach to the target and associate with factor B, which will be cleaved by factor D. This results in the formation of the C3 convertase C3bBb. The pathway continues with formation of the C5 convertase (C3bBb3b), cleavage of C5 and formation of MAC. Created with BioRender.com.

In neurological diseases, C1q was extensively studied for its role in synaptic pruning a process in which the degenerating synapse is stripped by microglia through a mechanism dependent on the complement C1q-C3 axis (Hong et al., 2016; Thion and Garel, 2018). The contribution of complement-mediated synapse pruning to neurodegeneration was first identified in a model of glaucoma in DBA/2J mice showing degeneration of retinal ganglion cells (Stevens et al., 2007). Later on other studies on human post mortem nervous tissues (Michailidou et al., 2015; Ramaglia et al., 2021) and experimental models of neurodegenerative diseases (Hong et al., 2016; Paolicelli et al., 2017; Michailidou et al., 2018) supported this finding and showed an effect of complement-mediated synaptic pruning on behavior (Lui et al., 2016), memory, and learning (Hong et al., 2016; Ramaglia et al., 2021). Now, a study by Zeng et al. suggested an involvement of the C1q-C3 axis in the degeneration of the human eye retina in high or pathological myopia. The authors reported significantly higher intraocular levels of the C1q-C3 axis proteins compared to control eyes, and a negative correlation between the amounts of C1q-C3 axis proteins and deep layer retinal thickness. Because complement is a major component of both the systemic and the neuro-inflammation, it can mediate neuroimmune actions in response to infections (Vasek et al., 2016). Hao et al. showed that complement-mediated synaptic pruning and neuroinflammation were boosted in Alzheimer's disease upon entrance of the periodontitis-causing pathogen *Porphyromonas gingivalis* (*Pg*) in the brain.

Next to its role in synaptic pruning, complement has additional roles in the pathology of the nervous system some of which are (partially) defined whereas, others not. In this Research Topic, Veremeyko et al. present data supporting a novel role for the C4B-encoded C4 protein in epilepsy. This role of C4 is associated with the expression of immediate early genes during an epileptic seizure and affects the cognition of mice receiving convulsant and subconvulsant doses of pentylenetetrazole.

In multiple sclerosis (MS), complement has an established role in demyelination (Prineas et al., 2001; Barnett et al., 2009). In particular, C1q has an antigen recognition-associated effector function that allows the efficient destruction of antibody-targeted myelin (Morgan et al., 2021). Notably, T cells and astrocytes located within a MS lesion respond to activated complement by increasing the expression levels of RGC32, a gene driving neuroimmune responses. A mini review by Tatomir et al. explains how RGC32 regulates astroglial cell reactivity to promote glial scar formation in a MS lesion.

In the gray matter, Evans et al. show that complement deposition and/or activation is associated with compartmentalized inflammation which is a driver of subpial cortical demyelination (Howell et al., 2011; Ahmed et al., 2022) and MS progression (van Olst et al., 2021). By examining human post-mortem MS brains the authors identified an association between the amounts of meningeal/subpial complement proteins and the extent of cortical demyelination. In addition, they reported an increased density of phagocytic C3a receptor (R) 1+ and C5aR1+ microglial cells/macrophages at the expanding edge of subpial and leukocortical lesions, suggesting a role for complement in the expansion of MS lesions.

Therapeutic agents developed to target the complement system carry a clear potential to alleviate diverse diseases including neurological diseases. A study by Seidel et al. demonstrated an immunomodulatory effect of the terminal complement pathway inhibitor BB5.1 in the brain of obese Ldlr^−/−^.Leiden mice. By means of immunohistochemistry and next generation sequencing, Seidel et al. showed that systemic administration of the BB5.1, a monoclonal antibody that blocks C5 cleavage, affected the microglial cell immunophenotype and modulated brain neuroinflammation in the obese mice. BB5.1 blocks the terminal complement pathway and is used in multiple animal studies as the equivalent of the anti-human C5 monoclonal antibody Eculizumab (Zelek et al., 2020). Lekova et al. from Cardiff University published an article characterizing a novel anti-complement inhibitor blocking the C7 protein of the terminal complement system. In this article they assessed the *in vitro* function, binding epitopes, and mode of action of three monoclonal antibodies targeting the C7 protein. The authors concluded that one of them, the TPP1820 mAb was effective in preventing experimental myasthenia gravis (MG) and provided a stratification assay for the detection of MG patients which are predicted to respond to an anti-C7 therapy. Last but not least in the group of therapy-related articles of this Research Topic, Li et al. moved their focus upstream in the complement cascade to study the response of human and murine macrophages bearing the C3a receptor, the cognate receptor for the complement peptide C3a, to the C3a antagonist TLQP-21. TLQP-21 is a neuropeptide derived from the VGF precursor protein. The authors confirmed the binding of TLQP-21 to C3aR but reported a low potency of the human peptide to activate the human primary macrophages concluding that a C3aR-dependent action of TLQP-21 on macrophages may not be physiologically relevant in humans.

This Research Topic show that the complement system is involved in more pathways than only combatting microbes. It has many more important functions in development and maintenance of a healthy nervous system.

## Author contributions

IM: Writing—original draft, Writing—review and editing. KF: Writing—review and editing. MB: Writing—review and editing. NG: Writing—review and editing. FB: Writing—review and editing.
